# All Is Good, but Not Really: Daily Reports of Satisfaction With Home- and Community-Based Services

**DOI:** 10.1093/geroni/igaa057.2194

**Published:** 2020-12-16

**Authors:** Jyoti Savla, Karen Roberto, Aubrey Knight, Rosemary Blieszner, Brandy Renee McCann, Emily Hoyt

**Affiliations:** 1 Virginia Tech, Blacksburg, Virginia, United States; 2 Virginia Tech Carilion School of Medicine, Roanoke, Virginia, United States; 3 Virginia Polytechnic Institute and State University, Blacksburg, Virginia, United States

## Abstract

An extensive body of literature documents correlates of and barriers to health service use, yet much less is known about satisfaction with home- and community-based services for persons with dementia (PwD). Daily diary data from 122 rural caregivers (CG) of PwD (814 daily diaries) were used to assess everyday service use experiences. At the last diary interview, CG identified areas where service use expectations were and were not being met. CGs reported problems with services used on fewer than 5% of study days (e.g., service provider was delayed because of car trouble). In contrast, 82% of CG identified areas where service expectations were not being met. Their most common concerns were lack of control over service availability and lack of adequate training among service providers. Recommendations for alternative ways for capturing service use satisfaction will be offered, and implications for theory and practice will be discussed.

